# The influence of *ROS1* fusion partners and resistance mechanisms in *ROS1*‐TKI‐treated non‐small cell lung cancer patients

**DOI:** 10.1002/1878-0261.70109

**Published:** 2025-08-29

**Authors:** Fenneke Zwierenga, Christa Dijkhuizen, Patrick Korthuis, Wim Timens, Harry Groen, Jeroen Hiltermann, Anke van den Berg, Lyndsay Drayer, Anthonie van der Wekken

**Affiliations:** ^1^ Department of Pulmonary Medicine University of Groningen, University Medical Center Groningen The Netherlands; ^2^ Institute For Life Science and Technology Hanze University of Applied Sciences Groningen The Netherlands; ^3^ Department of Pathology and Medical Biology University of Groningen, University Medical Center Groningen The Netherlands

**Keywords:** Non‐small cell lung cancer, resistance, ROS1

## Abstract

Clinical outcomes in *ROS1*‐fusion positive (*ROS1*+) non‐small cell lung cancer (NSCLC) by fusion partner and resistance mechanisms are limited. This cohort study included 56 *ROS1+* patients (FISH or NGS confirmed); fusion partners were identified in 27 cases, including *CD74* (*n* = 10), *EZR* (*n* = 7), and *SDC4* (*n* = 7). Clinical data were available for 50 patients (median age 62; 51% female; 32% never‐smokers). Forty patients received tyrosine kinase inhibitors (TKIs), mostly crizotinib (*n* = 38). Crizotinib showed a 55% objective response rate (ORR) and a median progression‐free survival (mPFS) of 5.3 months. Brain metastases (HR 2.65, 95% CI 1.06–6.60, *P* = 0.037) and prior chemotherapy (HR 3.17, 95% CI 1.35–7.45, *P* = 0.008) had a higher risk of progression. Sixteen patients received subsequent lorlatinib, with an ORR of 28% and mPFS of 3.7 months. G2032R and L2026M resistance mutations were identified in four lorlatinib non‐responders, and *in vitro* studies confirmed resistance to lorlatinib. Fusion partners did not affect crizotinib outcomes. Lorlatinib was ineffective against on‐target resistance. Real‐world data showed lower TKI efficacy than clinical trials, highlighting the role of clinical and molecular factors in treatment response.

## Graphical abstract

This real‐world study of *ROS1+* NSCLC highlights fusion diversity, treatment outcomes with crizotinib and lorlatinib, and *in vitro* experiments with resistance mechanisms. G2032R drives strong resistance to ROS1‐targeted TKIs, especially lorlatinib. Fusion partner location does not affect overall survival to crizotinib or lorlatinib. Findings support the importance of re‐biopsy and next‐generation inhibitors to overcome resistance to ROS1‐targeted tyrosine kinase inhibitors.

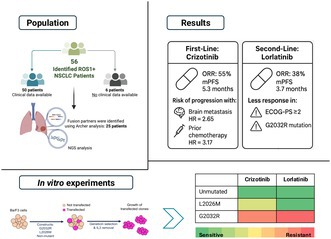

**Abbreviations**
BOR: best overall response
BSC: best supportive care
CD74: cluster of differentiation 74 (a *ROS1* fusion partner)
CI: confidence interval
CT: computed tomography
DoT: duration of treatment
ECOG‐PS: eastern cooperative oncology group performance score
ER: endoplasmic reticulum
FFPE: formalin‐fixed paraffin‐embedded
FISH: fluorescence *in‐situ* hybridization
G2032R: glycine 2032 to arginine substitution (mutation in *ROS1*)
IC50: half maximal inhibitory concentration
IL3: interleukin 3
L2026M: leucine 2026 to methionine substitution (mutation in *ROS1*)
MAPK: Mitogen‐activated protein kinase
NGS: next‐generation sequencing
NSCLC: non‐small cell lung cancer
OS: overall survival
PD: progressive disease
PFS: progression‐free survival
PR: partial response
Q2022P: glutamine 2022 to proline substitution (mutation in *ROS1*)
RECIST: response evaluation criteria in solid tumors
*ROS1*+: *ROS1*‐fusion gene positive
S1986F: serine 1986 to Phenylalanine substitution (mutation in *ROS1*)
SABR: stereotactic ablative body radiotherapy
SD: stable disease
TKI: tyrosine kinase inhibitor
UMCG: University Medical Center Groningen
WMO: Medical Research Involving Human Subjects Act (Netherlands)
WT: wild type

## 1 Introduction

*ROS1*‐fusion gene positive (*ROS1*+) non‐small cell lung cancer (NSCLC) accounts for ~2% of newly stage IV NSCLC adenocarcinoma patients. Identifying *ROS1* fusions is critical, as targeted treatments significantly prolong survival and enhance the quality of life compared to conventional chemotherapy [1]. Various tyrosine kinase inhibitors (TKIs), including crizotinib and lorlatinib, are effective in treating *ROS1+* NSCLC [2, 3, 4, 5, 6]. These TKIs bind to the ROS1‐fusion protein, inhibiting its phosphorylation activity on downstream proteins and suppressing tumor growth [6].

Over 30 fusion partners have been identified in *ROS1+* NSCLC, with *CD74‐ROS1* (~44%) and *EZR‐ROS1* (16%) being the most prevalent [7, 8]. *ROS1* breakpoints are typically located in exons 32, 34, and 35, resulting in long (exon 32) or short (exon 34 or 35) *ROS1* fusions [1]. Previous research has shown that the long *CD74*/*SLC34A2‐ROS1* fusion preserves the transmembrane regions of both *ROS1* and its fusion partners and is associated with a poor response to crizotinib treatment in patients with NSCLC [9].

Structural differences in *ROS1* fusions affect subcellular localization and downstream signaling pathways, influencing cell survival and proliferation [10]. For example, *SLC34A2*‐ and *SDC4*‐*ROS1* fusion proteins are localized on endosomes, where they activate the MAPK pathway. In contrast, the *CD74‐ROS1* fusion protein localizes at the endoplasmic reticulum (ER), which results in compromised MAPK [11, 12]. Previous studies suggest that certain fusions, such as *CD74‐ROS1*, are associated with shorter median progression‐free survival (mPFS) and median overall survival (mOS) compared to others [12, 13]. However, there is no consistent evidence supporting a correlation between specific *ROS1* fusion partners and survival. Other studies report no different survival durations across different *ROS1* fusion partners [2, 5]. Most data relate to crizotinib, with limited information on how fusion partners affect response to other TKIs.

Although patients with *ROS1*+ NSCLC initially respond well, the selective pressure exerted by TKIs inevitably leads to the emergence of resistance. Resistance mechanisms can be categorized as on‐target mutations within the *ROS1* kinase domain and off‐target mechanisms involving other genes or signaling pathways. On‐target mutations, such as those in the *ROS1* ATP‐binding pocket, include the common resistance mutation *ROS1* Gly2032Arg (G2032R) [7]. Off‐target resistance mechanisms may involve mutations in genes like *EGFR* or amplification of *MET*, leading to the activation of alternative survival pathways. Preclinical *in vitro* experiments on cell lines can provide valuable information on responses of on‐target mutations to different ROS1 TKIs. The Ba/F3 cell line is commonly used to test responses of different on‐target mutations or fusion partners to clinically available TKIs [14].

There is limited information on the effectiveness of TKIs against different on‐target resistance mutations and the potential influence of *ROS1+* fusion partners on treatment outcomes. This study aims to characterize the spectrum of fusion partners and resistance mutations in patients with *ROS1*+ NSCLC and to analyze treatment responses to different lines of TKI therapy using real‐world data and the Ba/F3 preclinical cell line model.

## 2 Material and methods

### 2.1 Study design and patients

This retrospective cohort study included patients with unresectable locally advanced or metastatic *ROS1+* NSCLC referred to the University Medical Center Groningen (UMCG) between March 2014 and May 2024. According to local guidelines, *ROS1+* fusions were identified by fluorescence *in‐situ* hybridization (FISH, genomic level), or using the Nanostring nCounter^®^ Analysis System, hereafter called “Nanostring” (transcript level) (from May 2019 on), and RNA‐based NGS approach, for example, Archer FusionPlex Lung kit (ArcherDX Inc, Boulder, CO), hereafter called “Archer” (from October 2021 onwards). Additional Archer analyses were performed on cases tested by FISH or Nanostring at diagnosis when archival formalin‐fixed, paraffin‐embedded (FFPE) material was available. External samples were obtained with local ethical approval.

Clinical data collected were sex, age, Eastern Cooperative Oncology Group performance score (ECOG‐PS), tumor stage and histology, molecular profile (including fusion partner and mutations) at baseline and throughout treatment. All were extracted from electronic health records. Outcomes analyzed included mOS (time from diagnosis until death or lost to follow‐up), mPFS (time from start antitumor treatment to detection of progressive disease [PD], death or lost to follow‐up), best overall response (BOR) and duration of treatment (DoT), as per RECIST version 1.1 [15]. Diagnostic imaging reports were compared to baseline (or to start treatment) CT scans to determine BOR and mPFS. This study was evaluated by the UMCG Central Ethics Review Board as a non‐WMO study (research register number 11080) on March 10, 2023 [16].

### 2.2 Cell lines and viability assays

Parental murine interleukin‐3 dependent pro‐B (Ba/F3, RRID:CVCL_0161) cells were purchased from DSMZ (ACC 300; Braunschweig, Germany) and maintained in RPMI 1640 (L0495; Biowest, Bradenton, FL, USA), supplemented with 10% FBS (S1860; Biowest) and 10% IL3‐conditioned medium. Ba/F3 cells were transfected with pcDNA3.4 constructs (GenScript) to express *SLC34A2‐ROS1* fusions (WT, L2026M, or G2032R) following the LONZA 4D‐NucleofectorTM protocol. After transfection, cells were selected with 1000 μg·mL^−1^ geneticin (11558616; Gibco™, Waltham, MA, USA), followed by a second selection of culture without IL3 at 400 μg·mL^−1^ geneticin (Fig. S1). Once stable after freezing and thawing, they were maintained without IL3 at 400 μg·mL^−1^ geneticin. *ROS1* fusion genes were confirmed by Sanger sequencing. All cell lines used in this study have been authenticated within the past 3 years. For authentication, a cell pellet from each line was sent to Eurofins Genomics in Germany. Authentication of the parental and SLC34A2‐ROS1‐expressing Ba/F3 cells was performed using highly polymorphic short tandem repeat (STR) profiling specific for mouse cell lines. Additionally, the identity of the SLC34A2‐ROS1 variant‐expressing cells was confirmed by Sanger sequencing following DNA extraction (BaseClear, Leiden, Netherlands). All experiments were conducted using cells that tested negative for Mycoplasma contamination at the time of analysis.

The constructed Ba/F3 cells were seeded in 96‐well plates (5000 cells per well) and incubated (37 °C, 5% CO_2_) with varying inhibitor doses for 72 h following a 24‐h recovery period. MTS was added, and the cell viability was assessed using the CellTiter 96 AQueous One Solution Cell Proliferation Assay (G3581; Promega, Leiden, Netherlands). Concentration response curves and IC_50_ values were analyzed using a nonlinear regression in GraphPad Prism 10 software.

### 2.3 Statistics

Descriptive statistics, including the median with interquartile range (IQR) and counts (*n*) with percentages (%), were used to summarize patient and tumor characteristics. PFS and OS were analyzed using the Kaplan–Meier method, and survival differences were calculated with the log‐rank test. The efficacy data cutoff date for these analyses was May 1, 2024. Associations between characteristics and survival outcomes were first assessed using a univariate stratified Cox regression model. Variables with a *P*‐value ≤ 0.15 in the univariate analyses were included in a multivariate stratified Cox regression model. A *P*‐value of 0.05 was considered significant for the multivariable analysis. Age and gender were included in the multivariable model as confounders. Fusion partners were categorized into *CD74‐ROS1* positive versus non‐*CD74‐ROS1* and breakpoint exon 32 (long) versus breakpoint exon 34 (short) groups to ensure an adequate number of events for the Cox regression model.

## 3 Results

### 3.1 Patient characteristics and treatment modalities

This study identified 56 *ROS1*+ NSCLC tumor samples over the past 10 years, with clinical data available for 50 patients. The median age was 62 years, 51% being female, and 32% being never smokers (Table 1). At lung cancer diagnosis, 4% had stage I, 8% stage II, 16% stage IIIA/B–IV treated with curative intent, and 72% stage IIIC/IV with palliative treatment intent. Forty patients had an ECOG‐PS of 0–1 at the time of diagnosis. Relapse to stage IV occurred in 64% of patients initially diagnosed at stages I‐III. Forty patients received crizotinib as first‐line TKI, with ongoing treatment in five patients. Second‐line TKI treatments included lorlatinib, with discontinuation primarily due to systemic progression. NGS was utilized to identify resistance mechanisms post‐TKI treatment.

**Table 1:** Patient characteristics at baseline. Data is presented as median [range] or *n* (%). Eight patients relapsed to stage IV disease from stage I (*n* = 1), stage II (*n* = 2) or stage III (*n* = 5). Five patients were still on treatment on first‐line TKI treatment (crizotinib, *n* = 4; other, *n* = 1). Extensive treatment information can be found in Fig. 1. *In one case, a patient with a distant metastasis was treated with curative intent due to stereotactic ablative radiotherapy on that metastasis. Abbreviations: ECOG‐PS, Eastern Cooperative Oncology Group performance score, TKI, tyrosine kinase inhibitor.

Characteristic *n* (% from total)	Patients (*n* = 50)
Age (median [range])	62 [34–81]
Sex (female)	29 (51)
Stage at lung cancer diagnosis	
Stage I	2 (4)
Stage II	4 (8)
Stage IIIA/B‐ IV* (curative intent)	8 (16)
Stage IIIC/IV (palliative treatment)	36 (72)
*‐‐‐‐‐‐‐‐‐‐‐‐‐‐‐‐‐‐‐‐‐‐‐‐‐‐‐‐‐‐‐‐‐‐‐‐‐‐‐‐‐‐‐‐‐‐‐*	*‐‐‐‐‐‐‐‐‐‐*
Relapse to stage IV (% from total stage I‐III)	9/14 (64)
Testing method at diagnosis	
FISH	32 (64)
Archer FusionPlex Lung kit	8 (16)
RNA‐NGS e.g., Nanostring nCounter^®^ Analysis Systems	9 (18)
DNA‐NGS e.g., TruSight Oncology 500 (TSO500)	1 (2)
ECOG‐PS at diagnosis	
0	19 (38)
1	21 (42)
2	7 (14)
> 3	3 (6)
Smoking status	
Current	3 (6)
Former	26 (52)
Never	16 (32)
Unknown	5 (10)
Brain metastasis	14 (28)
1–4 lines chemotherapy prior to TKI	12 (24)
TKI treatment	40 (80)
First line—crizotinib	38/40
First line—non‐crizotinib	2/40
*‐‐‐‐‐‐‐‐‐‐‐‐‐‐‐‐‐‐‐‐‐‐‐‐‐‐‐‐‐‐‐‐‐‐‐‐‐‐‐‐‐‐‐‐‐‐‐*	
Second line TKI (% from total first line TKI)	23/40 (58)
Second line—lorlatinib	16/35
Second line—non‐lorlatinib	7/35
Second line—other treatment	12/35

**Fig. 1:**
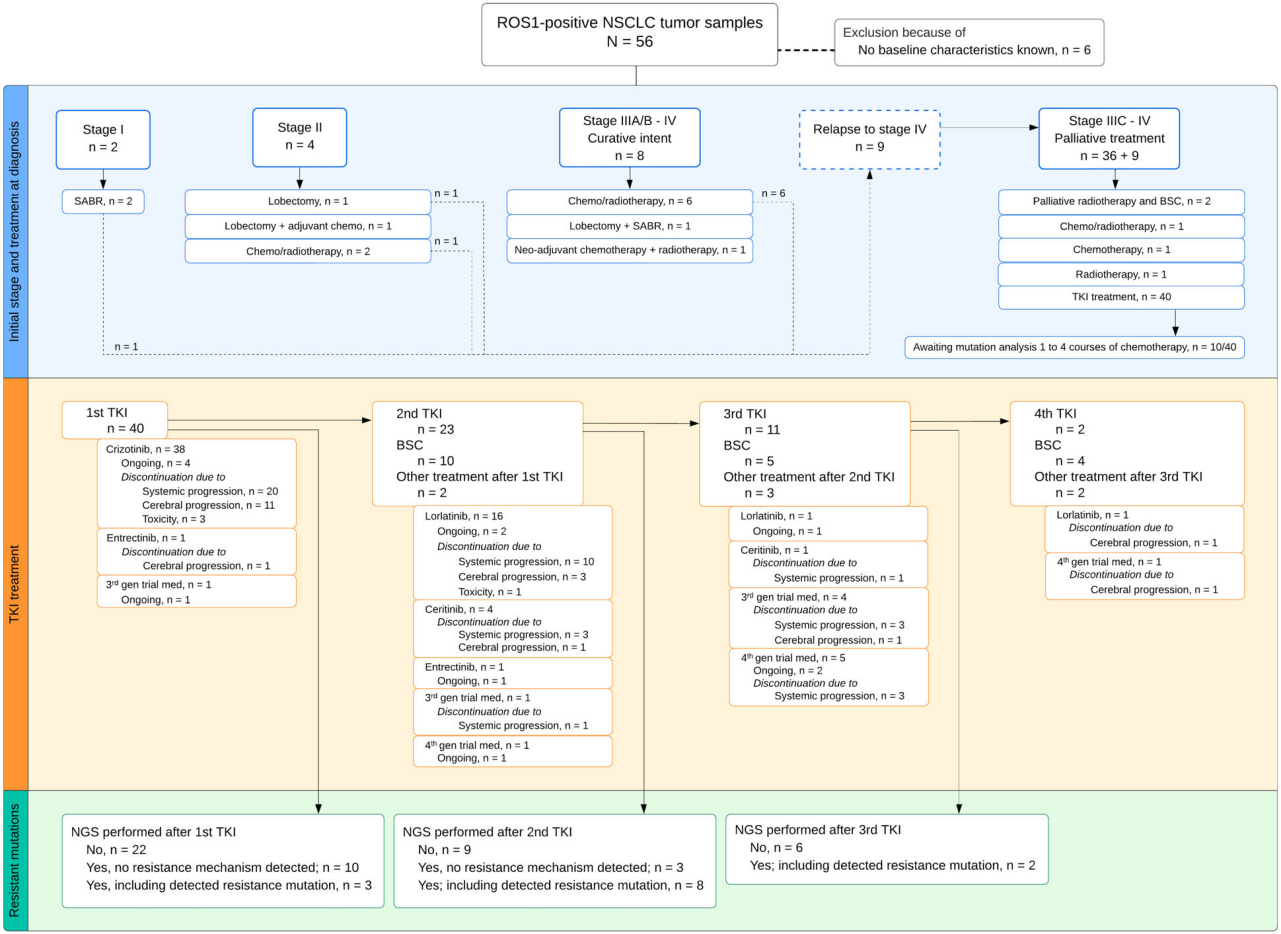
Patient staging and treatment modalities. The study analyzed 56 *ROS1*‐positive NSCLC tumor samples, excluding 6 patients due to unknown baseline characteristics and treatment modalities. The remaining 50 patients were classified by initial diagnosis stage and treatment approach. After the first TKI, NGS was conducted using the following methods: Archer (*n* = 7), Nanostring (*n* = 1), TSO500 (*n* = 1), and IonPGMv001 (*n* = 4). Following the second TKI, the methods used included Archer (*n* = 1), TSO500 (*n* = 6), Nanostring (*n* = 2), CHPv2 SOC‐1 (*n* = 1), and IonPGMv002B (*n* = 1). After the third TKI, NGS was performed using Archer (*n* = 1) and Nanostring (*n* = 1). Abbreviations: 3rd gen trial med, third‐generation trial medication; 4th gen trial med, fourth‐generation trial medication; BSC, best supportive care; *n*, number of patients; NGS, next‐generation sequencing; SABR, Stereotactic ablative radiotherapy; TKI, tyrosine kinase inhibitor.

Figure 1 summarizes staging and treatment of 50 *ROS1*+ NSCLC patients with available clinical data. Patients were grouped by initial stage and treatment. Relapse to stage IV occurred in 9/14 patients, from stage I (*n* = 1), stage II (*n* = 2), and stage III (*n* = 6). Five remained relapse‐free, with *ROS1+* status confirmed via surgery (*n* = 2) or biopsy (*n* = 3). Crizotinib was the first‐line TKI in 38 patients, with four patients still on treatment after a median of 2 years. Discontinuation reasons included distant (*n* = 20) or cerebral progression (*n* = 11) and toxicity (*n* = 3). Second‐line TKIs were given to 23 patients (lorlatinib in 16), with discontinuation due to distant (*n* = 10) or cerebral progression (*n* = 3) and toxicity (*n* = 1). Subsequent TKIs included lorlatinib, ceritinib, zidesamtinib, repotrectinib, and entrectinib, with discontinuation due to progression and other factors (Fig. 1). NGS identified resistance mechanisms post‐TKI treatment (*n* = 26) in tissue re‐biopsy in 22 patients, of whom 4 patients had undergone multiple biopsies after separate lines of therapy. The mOS (*n* = 50) was 25.5 months (95% CI 21.4–29.6).

### 3.2 TKI treatment and resistance mutations—first‐line treatment

**Fig. 2:**
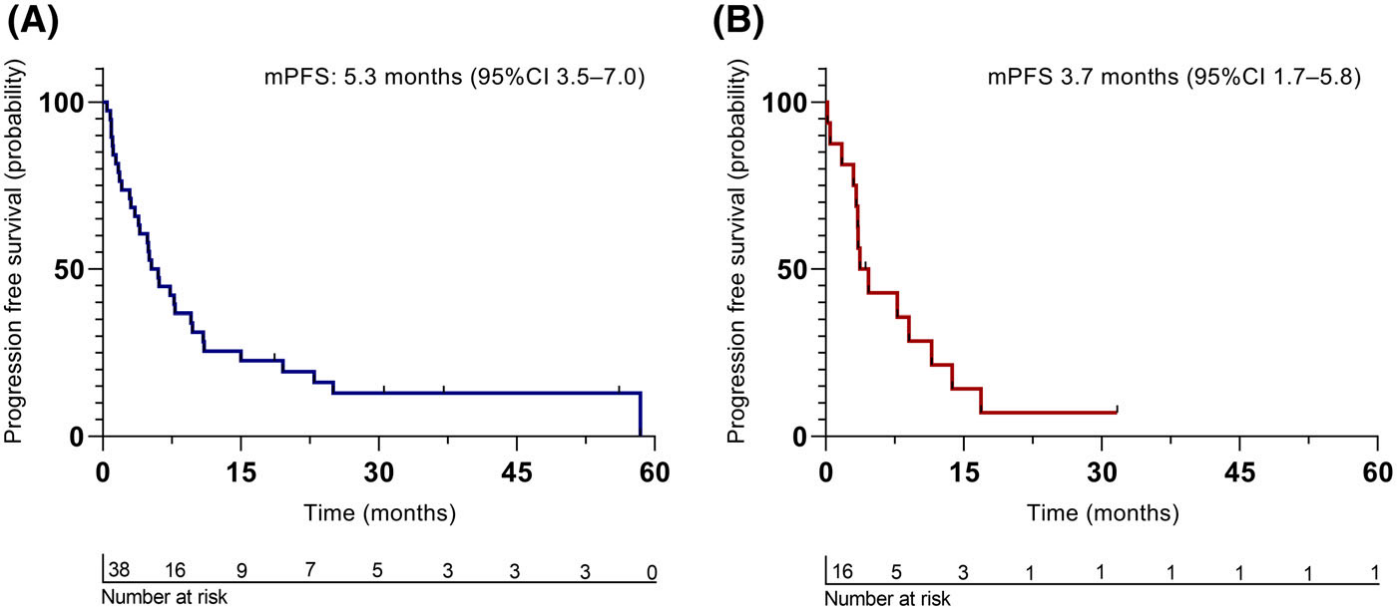
Progression‐free survival estimated with Kaplan–Meier method in 38 out of 50 ROS+ NSCLC patients who were treated with crizotinib (A) and 16 out of 23 patients who were treated with second‐line lorlatinib (B). Both curves show a rapid decline in PFS within the first few months, followed by a slower decline in survival probability over time. The number of patients at risk decreases across the time points, as shown below each graph.

Forty patients received first‐line TKI treatment (crizotinib; *n* = 38, non‐crizotinib; *n* = 2), of whom 12 patients received it after 1–4 lines of chemotherapy. Ten patients received non‐TKI treatments due to different stages of disease, as shown in Fig. 1. The objective response rate (ORR) of crizotinib was 55% with a mPFS of 5.3 months (95% confidence interval [CI] 3.5–7.0) (Fig. 2). Patients with brain metastasis at baseline (*n* = 9) had a significantly higher risk of progression (hazard ratio [HR] = 2.65, 95% CI: 1.06 to 6.60, *P* = 0.037). This was not associated with shorter mPFS (4 months [95% CI 3.6 to 4.4] versus 7.3 months [95% CI 3 to 11.6] *P* = 0.092). Those with prior treatment with chemotherapy (*n* = 11) also had a higher risk of progression (HR = 3.17, 95% CI: 1.35 to 7.45, *P* = 0.008) (Table 2), which was also associated with shorter mPFS (2.1 months [95% CI 0 to 4.1] versus 7.7 months [95% CI 4.7 to 10.8] *P* = 0.019). Four patients had both brain metastasis and prior treatment with chemotherapy. A more detailed overview of all treated patients (*n* = 40) is shown in Table S1.

**Table 2:** Subgroup analysis for progression‐free survival of crizotinib treatment (*n* = 38), conducted through both univariate and multivariate analyses. Abbreviations: CI, confidence interval; ECOG‐PS, Eastern Cooperative Oncology Group performance score; HR, hazard ratio. Significant values are indicated with an asterisk. Values significant enough in univariate analysis (*P*‐value ≤ 0.15) were included in the multivariate analysis. Age and gender were included in the multivariable model as cofounders (bold).

Characteristics	Univariate analysis		Multivariate analysis	
	HR (95% CI for HR)	*P*	HR (95% CI for HR)	*P*
Age	0.99 (0.97–1.02)	0.643	1.00 (0.97–1.03)	0.980
Gender	1.40 (0.69–2.82)	0.353	1.23 (0.59–2.57)	0.590
ECOG‐PS				
0	*Reference*			
1	1.52 (0.69–3.33)	0.300		
2	1.30 (0.46–3.69)	0.628		
Smoking history				
Never	*Reference*			
Former	0.91 (0.43–1.90)	0.793		
Current	0.42 (0.09–1.92)	0.263		
Brain metastasis	1.93 (0.86–4.34)	**0.113**	2.65 (1.06–6.60)	0.037*
Prior treatment chemotherapy	2.50 (1.14–5.49)	**0.022**	3.17 (1.35–7.45)	0.008*
Fusion partner *CD74‐ROS1*	1.81 (0.64–5.15)	0.263		
Fusion partner breakpoint exon 32 (long)	0.49 (0.15–1.62)	0.239		

**Fig. 3:**
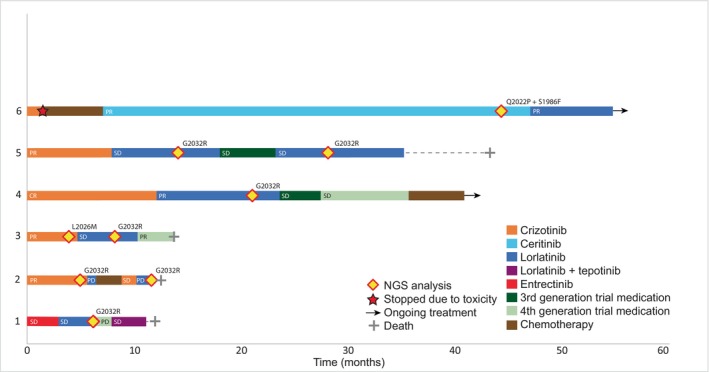
Detailed overview of six patients with on‐target resistance mutations, in combination with the duration and tumor responses of multiple lines of treatments. Four patients show no response (SD) to lorlatinib. In patient 4, the on‐target mutation G2032R is identified while a partial response to lorlatinib is recorded. After identifying the on‐target mutation, the patient becomes resistant to lorlatinib treatment. In patient 6, a partial response to lorlatinib was recorded after identification of Q2022P + S1986F. The distance between the two resistance mutations was too large to determine whether they are in cis or in trans. Given the differences between the two VAFs (c.6065A>C p.(Q2022P): 22% and c.5957C>T p.(S1986F): 5%), it is likely that part of the alleles only has the Q2022P variant and not the S1986F variant. Whether both variants are present in a subset of the tumor cells, or whether there are two distinct tumor cell populations remains unclear. Patient 1 was treated with a combination of lorlatinib and tepotinib due to the off‐target *MET* amplification. Abbreviations: CR, complete response; NGS, next‐generation sequencing; PD, progressive disease; PR, partial response; SD, stable disease.

NGS was performed at progression on first TKI for 13 out of 40 patients. No loss of the *ROS1* fusion target was detected in the patients who underwent re‐biopsy. For 27 patients, no subsequent NGS was performed at progression due to fast clinical deterioration (*n* = 7), ongoing treatment (*n* = 5), cerebral progression (*n* = 4), toxicity (*n* = 3), location not accessible for biopsy (*n* = 3), or low tumor percentage in the biopsy (*n* = 5). The 13 patients for whom NGS was performed all received crizotinib as first‐line TKI (Fig. 1). NGS revealed on‐target resistance mutations in two patients: G2032R (#2 in Fig. 3) and L2026M (#3 in Fig. 3). Both patients were subsequently treated with lorlatinib, but neither showed a tumor response.

### 3.3 TKI treatment and resistance mutations—second‐line treatment

Sequential TKI treatment with lorlatinib in 16 patients resulted in an ORR of 38% and a mPFS of 3.7 months (95% CI 1.7–5.8) (Fig. 2). Fifteen patients received lorlatinib as the next TKI after crizotinib and one after repotrectinib treatment. Out of 16 patients, 6 achieved a partial response (PR), 6 had stable disease (SD), and 4 experienced PD. The mPFS ranged from 0.2 months to 32 months (Fig. 2). At lorlatinib initiation, 11 out of 16 patients had brain metastasis. Seven had brain metastases prior to entrectinib/crizotinib treatment, while four developed brain metastases as a site of progression following crizotinib treatment. Five achieved a PR, five had SD, and one had PD as their BOR to lorlatinib. Of the five patients without brain metastasis, four did not respond to lorlatinib, and one patient had a PR. Thirteen of the 16 patients had an ECOG‐PS score of 0–1, and three patients had an ECOG‐PS score of 2. The three patients with an ECOG‐PS of 2 did not respond to lorlatinib, PD (*n* = 2) and SD (*n* = 1). Across the 13 patients with ECOG 0–1, responses varied from PD (*n* = 2), SD (*n* = 5), and PR (*n* = 6). Due to the small sample size, a reliable Cox regression could not be conducted.

Of the 23 patients receiving a second TKI, NGS data were obtained for 11 at the end of treatment. No loss of the *ROS1* fusion target was detected in the patients who underwent re‐biopsy. Reasons for not obtaining NGS data included fast clinical deterioration (*n* = 4), ongoing treatment (*n* = 3), low tumor percentage in the biopsy (*n* = 2), location not accessible for biopsy (*n* = 2), or cerebral progression (*n* = 1). In six patients, of whom five were treated with lorlatinib and one with ceritinib before biopsy, NGS revealed on‐target mutations: G2032R, *n* = 5; #1–5 and Q2022P + S1986F, *n* = 1; #6 in Fig. 3.

### 3.4 TKI treatment and resistance mutations—third‐line treatment

Eleven patients received a third TKI after progression/discontinuation of their second TKI, mostly 3rd or 4th generation trial medication. Due to rapid clinical decline, biopsies were limited; only three were performed, with two yielding results confirming retention of the *ROS1* fusion. One biopsy could not be analyzed due to a low tumor percentage. One patient had the G2032R mutation (#5 in Fig. 3). The other patient showed a MET amplification by FISH, with a MET to centromere ratio of > 2 as determined in nuclei of 50 tumor cells. In total, we found four off‐target alterations in three patients: *MET* amplification (*n* = 2), and *ERBB2* and *CCND1* (*n* = 1). One patient with *MET* amplification is shown in Fig. 3 as patient number 1. The other patient did not receive a *MET* inhibitor due to the rapid deterioration of condition due to lung cancer as the discovery of *MET* was after third‐line treatment.

A TP53 co‐mutation was observed 10 times in 7 different patients in different biopsies, that is, biopsy at diagnosis (*n* = 4), biopsy after first TKI (*n* = 1), and biopsy after second TKI (*n* = 5). No TP53 co‐mutation was found in a biopsy after third‐line treatment. Due to the small sample size, no associations with clinical features or survival could be made.

### 3.5 Analysis of fusion partners

The *ROS1* fusion partner was assessed in 31 out of 50 *ROS1*+ NSCLC patients with complete clinical data, while 6 additional patients without clinical data had available tumor samples for *ROS1* fusion partner evaluation. Archer analysis was not possible in 25 patients due to no tumor material left (*n* = 18) or poor RNA quality (*n* = 7). For 4 out of 31 patients, no *ROS1* fusion was detected, despite having a tumor cell percentage > 20%. For 27 patients, the RNA‐NGS analysis revealed a *ROS1* fusion transcript. Fusion partners were *CD74* (exon 34, *n* = 10), *EZR* (exon 34, *n* = 6), *EZR* (exon 32, *n* = 1) *SDC4* (exon 32, *n* = 7), *SLC34A2* (exon 32, *n* = 1), and the more uncommon fusion partners *LDLR* (exon 32, *n* = 1, PD on crizotinib with PFS of 3.5 months, no sequel treatment) and *HLA* (exon 34, *n* = 1, PR on crizotinib with PFS of 18.7 months and still ongoing). We did not find *de novo* alteration in patients diagnosed by RNA‐based NGS.

Univariate analysis available for patients with clinical data and available fusion partner showed no significant association between *CD74‐ROS1* positive (*n* = 7) fusions and non‐*CD74‐ROS1* (*n* = 14) fusion partners with the risk of progression during crizotinib TKI treatment (Table 2).

Furthermore, there was no significant difference in mPFS between patients with long (*n* = 9) versus short (*n* = 12) *ROS1* fusions in univariate analysis. Kaplan–Meier curve analysis, which included both groups, also revealed no significant difference in PFS between the groups (Fig. S2).

### 3.6 *In vitro* experiments

**Fig. 4:**
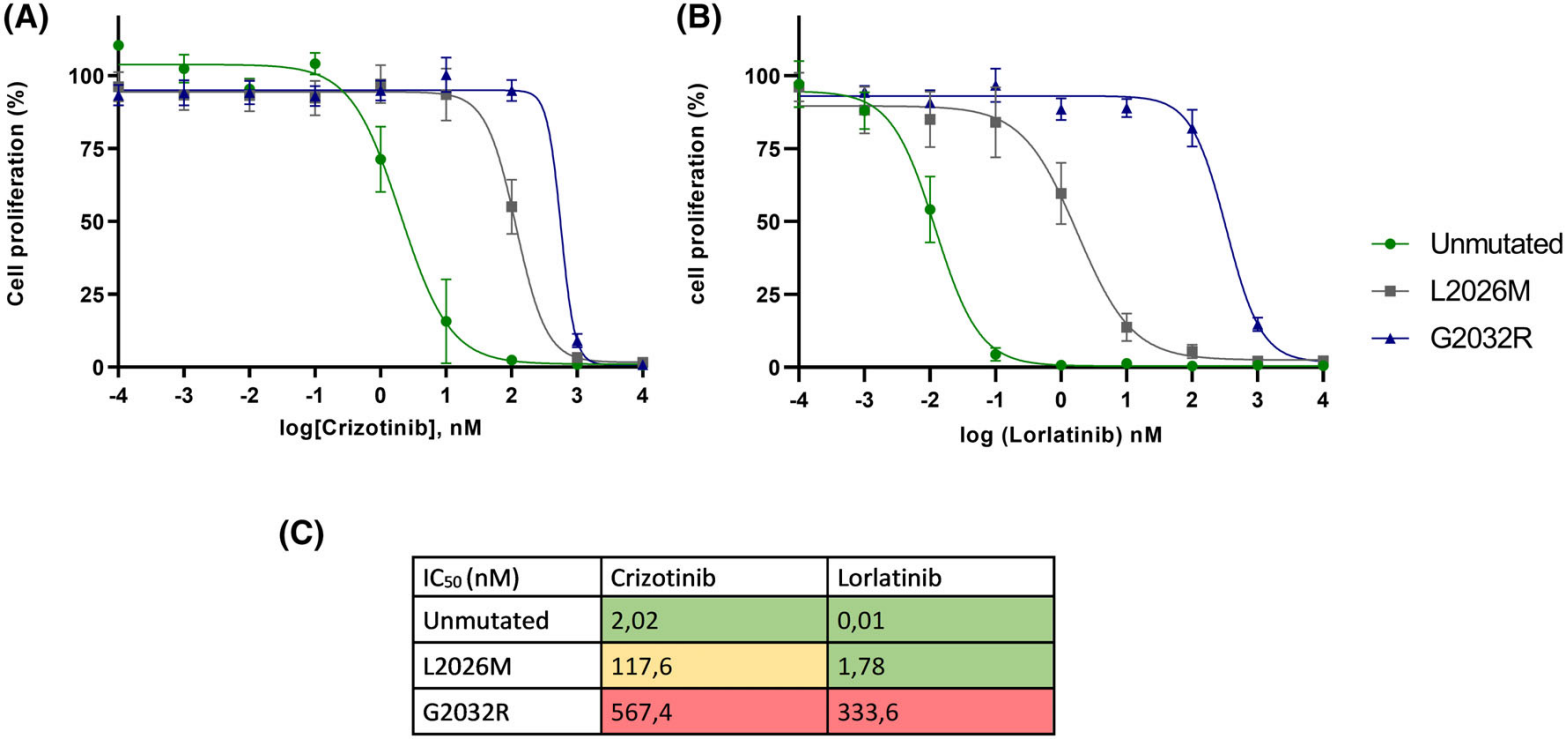
*SLC34A2‐ROS1* Ba/F3 cell (average ± SEM error bar) line with concentration response curves of crizotinib (A) and lorlatinib (B). Unmutated (green, *n* = 4), L2026M (gray, *n* = 8), and G2032R (blue, *n* = 8) expressing. The table (C) shows the associated IC_50_ values for comparison between crizotinib and lorlatinib against different mutation types. Green represents a lower IC_50_ representing the efficacy of the drugs on the mutation, orange intermediate IC_50_ and efficacy, and red high IC_50_ representing reduced efficacy.

*SLC34A2‐ROS1* constructs with and without the G2032R and L2026M mutations were transfected in Ba/F3 cells to determine the effect of crizotinib and lorlatinib treatment (Fig. 4). Ba/F3 cells with the unmutated fusion construct were more sensitive to lorlatinib compared to crizotinib, with a much lower IC_50_. The presence of the L2026M mutation reduces the efficacy of both drugs, with an intermediate response for lorlatinib. In contrast, the G2032R mutation leads to a > 200‐fold increase in IC_50_ value for crizotinib and > 20 000‐fold for lorlatinib. These results show a clear impact of both resistance mutations on drug efficacy *in vitro*, with the G2032R mutation presenting a substantial challenge for both crizotinib and lorlatinib.

## 4 Discussion

In this study, we analyzed *ROS1*+ NSCLC patients treated at an academic hospital over the past decade, focusing on clinicopathological features. Among 38 patients receiving crizotinib as the first TKI, the median PFS was 5.3 months (95% CI 3.5–7.0), with a higher risk of progression in patients with brain metastasis or prior chemotherapy. For 16 patients sequentially treated with lorlatinib, the mPFS was 3.7 months (95% CI 1.7–5.8). On‐target ROS1 resistance mutations were identified in six patients, including five with G2032R. Lorlatinib showed reduced efficacy in patients with G2032R or L2026M mutations. In Ba/F3 cells, lorlatinib activity was markedly reduced in G2032R mutants and modestly reduced in L2026M mutants compared to wild type. Analysis of ROS1 fusion partners in 31 patients revealed no significant association between fusion type or length and crizotinib response.

The efficacy of TKI treatment observed in this study is lower than in clinical trials prior to real‐world data. Crizotinib mPFS in previous reports ranged from 5.5 to 23.0 months, with longer durations in phase II trials (PROFILE 1001: 19.3 months; METROS: 22.8 months) and shorter in real‐world studies (8.6–13.1 months) [5, 17, 18, 19, 20]. Factors like ECOG‐PS ≥ 2, prior treatment, and baseline brain metastases are associated with shorter mPFS, consistent with our findings [21]. Crizotinib's limited CNS penetration may further explain poor outcomes in patients with brain metastases [22]. Another contributing factor to the low mPFS in our study may be false‐positive FISH results. Initially, ROS1 status was determined by break‐apart FISH, which is technically challenging and prone to error [23]. When excluding 17 patients with unvalidated FISH results (i.e., not validated by Archer analysis), the mPFS improved to 10.9 months (95% CI: 6.5–15.3). This suggests RNA‐based NGS, which offers higher specificity and fusion partner information, improves patient selection. All patients in the lorlatinib cohort had both FISH and NGS confirmation, reducing the risk of false positives. Finally, differences in study design, patient characteristics, and diagnostics may also explain the lower crizotinib efficacy observed.

The next‐generation *ROS1* inhibitor lorlatinib has shown clinical activity in crizotinib‐pretreated patients, with a 35% response rate and mPFS of 8.5 months in a phase 1–2 trial [4]. In a real‐world setting, lorlatinib has been evaluated as a second‐line TKI after crizotinib in several studies, like our cohort, in which the mPFS ranged from 7.6 to 9.7 months [24, 25, 26]. Our cohort reached a much lower mPFS of 3.7 months (range 0.2–31.7 months). Among 16 patients treated post‐crizotinib, response to lorlatinib varied. Patients with brain metastasis generally achieved SD or PR, supporting CNS activity with lorlatinib [4]. In contrast, patients with ECOG‐PS 2 had poorer outcomes, emphasizing the influence of performance status on treatment efficacy.

Besides lorlatinib, several next‐generation *ROS1* inhibitors are emerging, reflected in the varied treatments seen in our cohort (Fig. 1). Entrectinib showed an ORR of 67% and a mPFS of 15.7 months [27]. Repotrectinib achieved an ORR of 79% in TKI‐naïve and 38% in pretreated patients, with activity against the *ROS1* G2032R mutation [28]. Zidesamtinib, a macrocyclic TKI, also targets G2032R with limited TRK inhibition, offering efficacy in TKI‐refractory patients with brain metastases [29]. However, optimal sequencing of these TKIs remains undefined. Despite these advances, effective biomarkers for guiding treatment are still lacking. In our analysis, ROS1 fusion type did not significantly impact crizotinib outcomes. While Li *et al*. [9] reported significantly shorter mPFS with *ROS1* breakpoints in exon 32 (long) versus exon 34 (short), we observed the opposite trend. The G2032R mutation remains a key biomarker of resistance to lorlatinib, consistent with prior studies and our *in vitro* findings, which confirm lorlatinib's lack of activity against this mutation [4].

At the time of treatment, fewer next‐generation therapies were available, so re‐biopsies were done to confirm the *ROS1* fusion. All re‐biopsies were evaluated for phenotypic transformations per local pathological protocol (i.e., immunohistochemical staining to identify morphological and cellular changes such as epithelial–mesenchymal transition or small cell lung cancer–like transformation). We did not observe morphological changes in our re‐biopsies. Identifying resistance mechanisms is essential for selecting effective next‐line therapies. Newer TKIs, such as repotrectinib and zidesamtinib, show promise against on‐target mutations [28, 29]; while off‐target resistance may benefit more from mutation‐specific treatments, chemotherapy, or chemo‐immunotherapy [30]. In real‐world settings, re‐biopsies are very valuable to guide therapy after resistance.

We identified *ROS1* mutations L2026M and S1986F/Q2022P in two patients after first‐line TKI resistance. Clinical data on lorlatinib efficacy against S1986F, particularly combined with Q2022P, are limited, though one study and our findings suggest lorlatinib remains effective against S1986F post‐ceritinib [31]. Various *in vitro* experiments show lorlatinib has greater efficacy than crizotinib against L2026M [32, 33], but our clinical data do not support this [5]. Some limitations need to be noted regarding the present study. First, the small sample size and incomplete fusion partner data due to FISH‐based diagnosis in 64% of patients restrict the generalizability of the results. Extensive collaborations with other specialized cancer centers are required to increase the sample size and strengthen our findings.

## 5 Conclusion

Our findings underscore the need for clinicopathological data to guide TKI selection in real‐world settings. Key factors such as brain metastases, ECOG‐PS, and prior treatments should inform TKI choice. Crizotinib may be less suitable as first‐line therapy for patients with brain metastases. Ongoing phase III trials comparing crizotinib versus entrectinib (NCT04603807) and crizotinib versus repotrectinib (NCT06140836; TRIDENT‐3) will help define optimal treatment strategies. Lorlatinib appears ineffective against G2032R mutations but may be effective for S1986F, as supported by our data and prior studies. Further research is needed to understand the impact of different ROS1 fusions and support personalized treatment approaches.

## Conflict of interest

FZ has received consultancy fees from Janssen, CD has received a Dutch NOW Teacher grant, paid to the institution. PM has received a Dutch NOW Teacher grant, paid to the institution. WT has received incidental consultancy fees from Merck Sharp Dohme, Bristol‐Myers‐Squibb, and Altana, outside the submitted work and all paid to institution. In addition, he is a member of the Council for Research and Innovation of the Federation of Medical Specialists. JH has received grants or contracts from Roche, BMS, AZD, outside the submitted work and all paid to institution. He is a member of the committee “ter Beoordeling van Oncologische Middelen (BOM).” AW has received grants or contracts from AstraZeneca, Boehringer‐Ingelheim, Pfizer, Roche, and Takeda, has received consulting fees from AstraZeneca, Janssen, Lilly, Roche, and Takeda, has received payments or honoraria from AstraZeneca, BMS, Lilly, Pfizer, and Roche, has a leadership or fiduciary role in the oncology section NVALT, guideline committee NSCLC and CUP, dure geneesmiddelen committee NVALT and FMS, all outside the submitted work, and payments were to the UMCG. All other authors declare no conflict of interest.

## Author contributions

Conceptualization: FZ, CD, AB, AW. Data curation: FZ, CD, PK, AB, AW. Formal analysis: FZ, CD, PK. Validation: AB, LD, AW. Supervision: AB, LD, AW. Interpretation of results: FZ, CD, PK, WT, HG, JH, AB, LD, AW. Investigation: FZ, CD, PK, WT, HG, JH, AB, LD, AW. Visualization: FZ, CD. Methodology: FZ, CD, PK, AB, LD. Writing‐original draft: FZ. Project administration: FZ, CD, WT. Writing‐review and editing: FZ, CD, PK, WT, HG, JH, AB, LD, AW.

## Peer review

The peer review history for this article is available at https://www.webofscience.com/api/gateway/wos/peer‐review/10.1002/1878‐0261.70109.

## Supporting information


**Fig. S1.** Generation of independent Ba/F3 cell lines overexpressing SLC34A2‐ROS1 with on‐target mutations.
**Fig. S2.** Kaplan–Meier curve analysis revealed no significant difference in progression‐free survival between the groups regarding fusion types.

**Table S1.** Detailed overview of all treated patients (*n* = 40) with TKIs, also shown in Fig. 1. Abbreviations: BOR, best overall response; BSC, best supportive care; chemo, chemotherapy; CR, complete response; fig, figure; NA, not applicable; PD, progression of disease; PD, progressive disease; PFS, progression‐free survival; PR, partial response; SD, stable disease; TKI, tyrosine kinase inhibitor.

## Data Availability

The data that support the findings of this study are not publicly available due to ethical restrictions and the sensitive nature of patient information.
