# Supplementary material for: The influence of ROS1 fusion partners and resistance mechanisms in ROS1‐TKI‐treated non‐small cell lung cancer patients
Source: Mol Oncol. 2025 Aug 29;19(11):3023–34. doi: 10.1002/1878-0261.70109 (PMC12591307; doi:10.1002/1878-0261.70109)
Supplement: Supplementary file 1 — Fig. S1. Generation of independent Ba/F3 cell lines overexpressing SLC34A2‐ROS1 with on‐target mutations. Fig. S2. Kaplan–Meier curve analysis revealed no significant difference in progression‐free survival between the groups regarding fusion types. [file MOL2-19-3023-s002.docx]

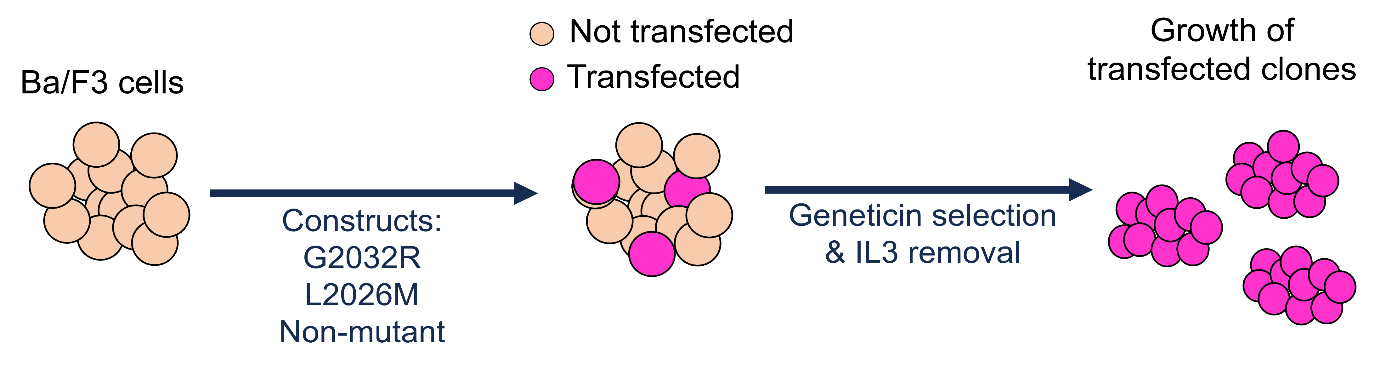
**Supplementary Figure 1** - Generation of independent Ba/F3 cell lines overexpressing SLC34A2-ROS1 with on-target mutations.

**Supplementary Figure 2** - Detailed overview of all treated patients (n=40) with TKIs, also shown in Figure 1. (A) Patients with CD74-ROS1 positive fusions versus non-CD74-ROS1 fusions did not show a significant difference in progression-free survival (log-rank, p=0.257). (B) Similarly, there was no significant survival difference in progression-free survival between breakpoint exon 32 (long) vs. breakpoint exon 34 (short) groups (log-rank, p=0.231).
